# Merging Brain-Computer Interface P300 speller datasets: Perspectives and pitfalls

**DOI:** 10.3389/fnrgo.2022.1045653

**Published:** 2022-12-21

**Authors:** Luigi Bianchi, Raffaele Ferrante, Yaoping Hu, Guillermo Sahonero-Alvarez, Nusrat Z. Zenia

**Affiliations:** ^1^Dipartimento di Ingegneria Civile ed Ingegneria Informatica, Tor Vergata University, Rome, Italy; ^2^Department of Electrical and Software Engineering, University of Calgary, Calgary, AB, Canada; ^3^Institute for Biological and Medical Engineering, Pontificia Universidad Católica de Chile, Santiago, Chile

**Keywords:** fair principles, BCI, P300, speller, dataset, database

## Abstract

**Background:**

In the last decades, the P300 Speller paradigm was replicated in many experiments, and collected data were released to the public domain to allow research groups, particularly those in the field of machine learning, to test and improve their algorithms for higher performances of brain-computer interface (BCI) systems. Training data is needed to learn the identification of brain activity. The more training data are available, the better the algorithms will perform. The availability of larger datasets is highly desirable, eventually obtained by merging datasets from different repositories. The main obstacle to such merging is that all public datasets are released in various file formats because no standard way is established to share these data. Additionally, all datasets necessitate reading documents or scientific papers to retrieve relevant information, which prevents automating the processing. In this study, we thus adopted a unique file format to demonstrate the importance of having a standard and to propose which information should be stored and why.

**Methods:**

We described our process to convert a dozen of P300 Speller datasets and reported the main encountered problems while converting them into the same file format. All the datasets are characterized by the same 6 × 6 matrix of alphanumeric symbols (characters and numbers or symbols) and by the same subset of acquired signals (8 EEG sensors at the same recording sites).

**Results and discussion:**

Nearly a million stimuli were converted, relative to about 7000 spelled characters and belonging to 127 subjects. The converted stimuli represent the most extensively available platform for training and testing new algorithms on the specific paradigm – the P300 Speller. The platform could potentially allow exploring transfer learning procedures to reduce or eliminate the time needed for training a classifier to improve the performance and accuracy of such BCI systems.

## Introduction

**Figure 1:**
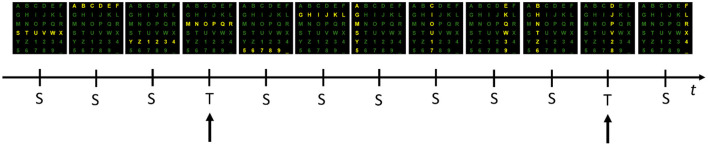
One example sequence of flashing rows and columns. Each of six rows and six columns flashes once while a user attends to the “P” character. Flashes of the 3rd row and 4th column containing the target (T) stimuli (i.e., “P”) evoke a response that can be measured in the EEG as the P300 component. Flashes relative to the 10 standards (S) do not evoke this response.

**Figure 2:**
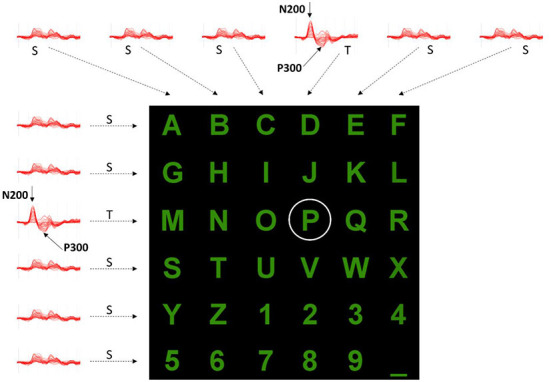
Example of evoked potentials observed after each row and column flashing while a user wanted to select the “P” character. The elicited responses after the 3rd row and 4th column Target (T) stimulations differ from those of other rows and columns (S, Standard). Positive y-axis directed downward.

Even after three decades of intensive research, most brain-computer interface (BCI) experiments are conducted in isolated and autonomous laboratories using proprietary software. These circumstances usually result in small data sets collected with different file formats. The IEEE-SA P2731 Working Group (Easttom et al., 2021) seeks to overcome that limitation and demonstrate the importance of having standard file formats and repositories, allowing easy sharing of data and software tools. This study aims to describe our process of combining datasets from several BCI experiments, which have the same paradigm and possess minimal differences. For this purpose, we chose a typical P300 Speller paradigm because several datasets are widely available in the public domain. The paradigm is characterized by the same 6 × 6 matrix of alphanumeric symbols (characters and numbers), arranged as depicted in Figures 1, 2.

Furthermore, all datasets are collected by the same subset of acquired signals (8 EEG sensors at the same recording sites). The combination would allow testing of different classifiers and machine learning (ML) algorithms, investigating error distribution over the grid of the matrix and answering meaningful questions (e.g., are errors uniformly distributed or distinctly observable over the edges of the grid?), evaluating different filtering procedures in the time or space domains, etc. We reported some significant differences in the acquisition and processing methods of the individual datasets and the main problems encountered during their conversion to an adopted file format. The conversion involved nearly a million stimuli (e.g., flashing rows and columns), which combined about 7 thousand selections made by 127 individuals. Our future work will extend the adopted format and conversion process to other datasets, relaxing certain requirements but storing all necessary information in data files. This would allow automating analysis methods through reusable software tools. Therefore, this study is a starting point (or a proof-of-concept) of the need for a BCI standard file format and large-scale datasets.

The P300 is an event-related potential (ERP) elicited in the decision-making process and reflects processes involved in stimulus evaluation or categorization (Sutton et al., 1965). It can be elicited using an oddball paradigm, in which low-probability target items are mixed with high-probability standard (or non-target) items. It manifests as a positive deflection in voltage with a latency of about 300 ms. Farwell and Donchin (1988) exploited the P300 component to create a system that allows people to make selections from items displayed as individual elements of a matrix and to use those selections to communicate without using the natural pathways of nerves and muscles (Wolpaw et al., 2000) but just their electrical brain activity. This communication system is called P300 Speller.

In such a paradigm, a user is positioned in front of a computer screen where symbols (such as characters and numbers) are displayed, as shown in Figures 1, 2. Then, entire rows and columns start flashing in a pseudo-randomized order while the user is attending to a specific alphanumeric character. Figure 1 illustrates an example of one sequence of 12 stimuli in which all rows and columns flash only once. If the user is attending to the letter “P”, then a P300 response should be elicited by flashing the fourth and eleventh target (T) stimuli but not by the other 10 standards (S) stimuli. Other than the P300, another component called the N200 – a negative deflection with a latency of around 200 ms – is elicited by visual stimulation.

By analyzing the responses after each row and column flashing, as indicated in Figure 2, it is possible to identify which row and column contain the P300 component and then, at their interception, determine the character the user wants to select. However, several iterations should be provided for each character selection as the signal-to-noise ratio is low, and some processing (e.g., averaging, voting) could improve P300 (and N200) detection. Following the conventions of neurophysiology, the positive y-axis is directed downwards.

ML algorithms are extensively used to identify the P300 component. The identification needs a data-based training procedure to recognize the elicited brain activation. This procedure, called calibration, is sometimes repeated for each subject (i.e., the user) having dedicated recording sessions, implying that a BCI system cannot be utilized immediately and requires some time to be configured. Because of inter- and intra-subject variability, the calibration may last significantly long.

In the last decades, the original P300 Speller paradigm from Farwell and Donchin (1988) was replicated in many experiments (Krusienski et al., 2008; Guger et al., 2009; Aricò et al., 2014; Lu et al., 2020) and, in some cases, collected data were released to the public domain. The freely available data allows several research teams, particularly those in the field of ML, to develop and test various new techniques to improve users' communication speed and system stability.

During the last years, the availability of larger datasets has allowed several advances in the matter of automatic classification by, specifically, enhancing Deep Learning (DL) capabilities (Bengio et al., 2021). Furthermore, it is widely accepted that ML algorithms need a significant amount of data to achieve high-accuracy results (Alzubi et al., 2018) and that data collection is a critical step for different communities when developing classification models (Roh et al., 2019). Therefore, it is expected that by collecting and mixing data from other labs, the training procedures of ML algorithms for BCI systems could be improved by reducing or even eliminating the P300 calibration and increasing the overall system performance. However, the main obstacle to creating large databases is that all public datasets are released in different file formats, most likely those used by the contributors. This occurs because there is no standard way to share them. Additionally, almost all public datasets need humans to read documents and/or scientific papers to retrieve relevant information, preventing software automation. Currently, datasets must be converted into a unique file format for merging and processing. This procedure is time-consuming as different software tools must be implemented for each of the datasets, and several documents/papers must be read to retrieve the desired information. An example attempt to enhance accessibility to different BCI datasets is the MOABB project (Jayaram and Barachant, 2018) which also includes P300 datasets collected from varying experiments but in different settings.

Within the scope of this study, we adopted a unique file format to demonstrate the importance of having a standard and to propose which information should be stored and why, following the ongoing activity of the IEEE-SA P2731 Working Group (Easttom et al., 2021). We then described our process of converting 11 datasets of P300 Speller. All the datasets are characterized by the same 6 × 6 matrix of alphanumeric symbols, as depicted in Figure 2, due to being the most popular setting in P300-based BCIs and by the same subset of acquired signals (8 EEG sensors at the same recording sites: Fz, Cz, Pz, Oz, P3, P4, PO7, and PO8).

The entire process could be easily extended to datasets of other P300 paradigms, even non-matrix-based ones with sufficient definitions of a mapping between stimuli and actions toward an external environment (e.g., character selection, demotics, wheelchair control, etc.). For simplification, we focused, however, on the datasets derived from the same matrix of the P300 paradigm with the same set of EEG sensors. We reported the main problems relating to their conversion into the adopted file format and the main differences in their acquisition and processing methods. We converted nearly a million stimuli (e.g., flashing rows and columns) relative to more than 6800 symbols spelled by 127 subjects. The converted files constitute the most extensive available BCI P300 Speller database, which can be used to train and test new ML algorithms later. This could allow exploring transfer learning approaches, which store knowledge gained when training one or more subjects and apply the knowledge to a different population of users. Moreover, this could reduce or eliminate the time necessary to train an ML algorithm at every recording session and improve the systems' performance and accuracy. The study also aims to illustrate the main variations among the datasets and describe some difficulties in retrieving information necessary for processing them. Such description would provide insights to propose standards that adhere to the FAIR principles (Findability, Accessibility, Interoperability, and Reuse of digital assets) (Wilkinson et al., 2016) and demonstrate their relevance. Indeed, the lack of standards implies that each team distributes its datasets according to its internal format. This could lead researchers with different backgrounds to interpret the same information at various levels of relevance, meaning, and even importance.

Finally, it is important to underline that this study should not be considered a competition among the various teams that generously provided the opportunity of analyzing their datasets. Instead, we would like to thank all research teams that shared their datasets to make this study possible and encourage others to do the same to contribute to the growth of the BCI research field.

## Methods

To create a unique database by aggregating datasets from several experiments, the entire process was divided into 6 main phases:

1) the identification of the information to be stored in the data files of datasets;
2) the retrieval of P300 Speller dataset candidates suitable for the study;
3) the exclusion of datasets from which all the information defined in 1) cannot be retrieved;
4) the definition of a unique file format for holding all data related to BCI;
5) the implementation of software tools to convert all datasets into the defined format;
6) the development of software tools to retrieve and visualize information from the datasets in the defined format.

### The identification of the information to be stored in files

The IEEE Standard Associations P2731 levels 0 and 1 specifications for BCI data storage were adopted (Bianchi et al., 2021). They deal with the information that should be stored in a file and not the technology to be used or any software implementation. Although not yet standardized, the specifications represent a work in progress and are used as a proof of concept for this study. According to level 1, all the information necessary to train a ML algorithm must be stored in a file. This file should include all acquisition parameters (level 0), the characters to be spelled, the encoder, the generated stimuli (e.g., which row or columns flashed), the target and standard stimuli and the timing of the beginning and end of each stimulation sequence for selecting a character. At this proof-of-concept stage, we omitted demographic information because some datasets contain no such information or describe the information poorly. For example, the language of the subjects/patients could be deduced from the spelled words in some cases but not in others. Furthermore, the age and sex of subjects/patients are sometimes missing from datasets.

#### Data acquisition

As a subset of level 1, the IEEE P2731 level 0 specifications were used for data acquisition. Besides the signals, the level 0 specification includes the required reference and ground locations, sensor labels, sampling rate, and events such as delivered stimuli. In the P300 Speller paradigm, the delivered stimuli (i.e., the flashing rows and columns) are synonyms of logical symbols (LS), while selected alphanumeric characters are the semantic symbols (SS). Surprisingly, sensor labels are often missing in data files but provided in additional documents and/or papers, which also hold other information regarding the used EEG apparatus and the electrode technology (e.g., active vs. passive, dry vs. wet, etc.).

#### Pre-processing

The knowledge of what pre-processing the signals underwent before their release is essential. As noted in Sahonero-Alvarez et al. (2021), filtering in the frequency domain is mainly used, as well as spatial domain filters such as Independent Component Analysis (also allowing for identification and removal of noise sources) or Laplacian filtering. Due to the easy replication of most pre-processing methods, sharing the signals un-processed would be a good practice to minimize pre-processing methods' implementation effects and type (e.g., IIR vs. FIR filters). Furthermore, signals are sometimes stored as short and segmented epochs, one for each stimulus and of fixed duration, which can be too short as compared to other datasets, thus preventing their comparison. Moreover, if the interval across two consecutive stimuli is smaller than the segmented epoch length, as in most cases, data segments partially overlap, and then some samples are stored several times, thus causing redundancy and larger files to be downloaded.

#### The encoder: Classifiers

The logical alphabet for a given P300 system may be defined according to the IEEE P2731 BCI functional model as the outputs of the transducer (Bianchi et al., 2021), which oversees the acquisition and processing of brain signals (Sahonero-Alvarez et al., 2021). In most cases, the logical alphabet is equivalent to the set of possible classifier labels (e.g., the 6 rows and the 6 columns) and is used to solve ML problems. Such outputs are computed, along with the pre-processing stage, by executing classification tasks and are used by the encoder to obtain the semantic alphabet: sequences of LS are mapped to a SS (e.g., the pair Row3 Col4 maps the “P” character). This mapped information is used to allow spelling. In practice, when authors propose a given BCI system, they also describe the implemented encoding that comprises a set of algorithms for classification. Some common classifiers for P300, according to Wang et al. (2018) and Abiri et al. (2019) are Linear Discriminant Analysis, Bayesian Regression Analysis, Stepwise Discriminant Analysis, Support Vector Machines, and Artificial Neural Networks, although, in recent years, there has also been an important interest in Deep Learning based classifiers (Aggarwal and Chugh, 2022). In any case, whichever the classifier is, the parameters to reproduce findings and results are completely required. Unfortunately, this information is not always made available as it implies that authors should upload and publish their code and models as well as their configurations.

#### Paradigm

The following parameters of the P300 speller paradigm were stored in the converted files:

1) The Semantic Alphabet (SA): it is the set of 36 alphanumeric characters (the Semantic Symbols, SS) that can be spelled (see Figure 2).
2) The Logical Alphabet (LA): it is the set of labels representing the possible outputs of a classifier. To each of the 12 labels (i.e., the 6 rows and 6 columns) corresponds a Logical Symbol (LS) and vice versa. LSs and labels can then be used interchangeably.
3) The Encoder: it defines the mapping between sequences of SSs and LSs. For example, the SS “P” is generated after the classification of the LS pair “Row 3” and “Col 4”.
4) The Inter Stimulus Interval (ISI): it is the interval between two stimuli in a flashing sequence, such as 12 flashes in Figure 1. The ISI, typically of the order of hundreds of milliseconds, can be deducible from the stored stimuli events.
5) The number of iterations for each spelled character: minimum and maximum values are provided as the number can vary within a recording session. Like the ISI, the number can usually be deduced from the stored events.

### The retrieval of P300 speller datasets

To retrieve the P300 Speller datasets, we first identified leading online data repositories through a Google search and then looked at each repository, which led to the following list: BNCI Horizon 2020 (2015) (http://bnci-horizon-2020.eu), Zenodo (2013) (https://zenodo.org), IEEE Data Port (https://ieee-dataport.org/datasets), Kaggle (2010) (https://www.kaggle.com/datasets), GigaDB (2011) (http://gigadb.org), OpenNeuro (2011) (https://openneuro.org), and Figshare (https://figshare.com).

Then, we searched P300 BCI datasets within each of the selected repositories. The keywords for the search included: “BCI”, “Brain-Computer Interface”, and “P300”. One minor problem yielded by the search was the presence of several false positives due to different meanings of the “BCI” acronym in other disciplines (e.g., genetics, biology), which was easily solved by manual screening. Another problem was the impossibility of *a-priori* assessing the quality of the various datasets. After downloading and inspecting these datasets, we excluded small datasets (<5,000 stimuli, which limit the training power procedure) or low-quality (noisy) recordings, as judged by a skilled neurophysiology technician.

Moreover, we checked the datasets for their concurrent fulfillment of two criteria: the adoption of the same 6 × 6 alphanumeric symbols matrix as depicted in Figure 2 and the possibility, after the conversion, of being IEEE P2731 level 1 specification compliant (to provide the ability to train a classifier) without asking for missing information to donors or repositories administrators. The datasets meeting the criteria formed a short list for consideration, as indicated in Table 1. The links to download the datasets were also included in the same table. Finally, the list was further expanded, as shown in Table 1, thanks to BCI colleagues/researchers who contributed with some datasets satisfying the criteria mentioned above.

**Table 1:** List of datasets for consideration and their origines.

**Repository**	**DataSet**	**Short name**	**Link**
BCI competition 2	iib	BCI Comp 2	https://www.bbci.de/competition/ii/
BCI competition 3	ii	BCI Comp 3	https://www.bbci.de/competition/iii/
BNCI Horizon 2020	8	BNCI DS8	http://bnci-horizon-2020.eu/database/data-sets
BNCI Horizon 2020	9	BNCI DS9	http://bnci-horizon-2020.eu/database/data-sets
BNCI Horizon 2020	12	BNCI DS12	http://bnci-horizon-2020.eu/database/data-sets
Kaggle	Akimpech	Akimpech	https://www.kaggle.com/datasets/electrototo/akimpech
braINterface	P300 180	BTFC 180	https://www.brainterface.com/joomla2/downloads/category/3-data
braINterface	P300 250	BTFC 250	https://www.brainterface.com/joomla2/downloads/category/3-data
braINterface	P300 800	BTFC 800	https://www.brainterface.com/joomla2/downloads/category/3-data
IEEE DataPort	P300 BCI	ERP-P300	https://ieee-dataport.org/documents/event-related-potentials-p300-eeg-bci-dataset
IEEE DataPort	GIB-UVA	GIB-UVA	https://ieee-dataport.org/documents/gib-uva-erp-bci-dataset

### Excluded datasets

Dataset 12 of the BNCI Horizon 2020 (Guger et al., 2009) was excluded due to its lack of sorting information for the sensors used. Although the sensors were illustrated in documentation with the dataset and the high quality of the recordings, it is not feasible to determine a correspondence between an EEG sensor and its recording signals. After all, one of the criteria prohibits extra information requests from contacting the dataset's contributors.

### Defining a unique file format

The IEEE P2731 proposal (Bianchi et al., 2021) follows the FAIR principles (Wilkinson et al., 2016; Stall et al., 2019) but does not deal with adopting technologies for data storage. Extensible Markup Language (Bray et al., 2008) and JavaScript Object Notation (JSON, 1999) are possible technologies for real implementation, widely utilized in several ICT areas because they are human-readable, extensible, and easy to understand and use. Based on XML, the NPX file format (Bianchi et al., 2007) was adopted for this study. The format and its associated software tools – the NPXLab Suite (Bianchi, 2018) – were implemented by one of the authors of this manuscript and had already been used in several studies. All datasets listed in Table 1 were then converted into the NPX file format. The conversion added structured information with backward compatibility, meaning that adding new data in the future will not break the existing software that today supports it. Software tools (the NPXLab Suite) are freely available for reviewing and analyzing EEG, MEG, and ERP and converting and exporting data. Furthermore, NPX files can also be imported into MATLAB and converted into other formats. Caution is needed during conversion from NPX into other supported formats, as some information could be lost because destination formats might not allow storing some BCI-related data.

### Implementation of file conversion software tools for all datasets

The subsections below describe each dataset in Table 1, including the problems we encountered in retrieving the necessary information to generate its valid IEEE P2731 level 1 file and converting it into the adopted NPX file format. The sources for retrieving the necessary information are summarized as follows:

1) Data File (DF): this is usually a MATLAB or a binary file, which stores most of the acquisition parameters and settings. The formatted nature of the file allows for automating certain operations *via ad hoc* software implementations.
2) Scientific Publication (SP): the publication is commonly a downloadable PDF file containing some study details in the Materials and Methods section. Humans need to read the publication to extract the necessary information.
3) Descriptive Paper (DP): The paper is conventionally a PDF or TXT file, which explains in detail the file format and structures used to represent some entities (e.g., sensors, events, markers, etc.) of a study. A programmer must read the paper to implement software tools for extracting the necessary information.
4) Formatted Text Files (FTF): They are generally TXT files that store, for example, sensor labels and coordinates or other elements such as words to be copied during a calibration session, number of iterations, etc. The formatted nature of the file allows for automating certain operations.

Table 2 gives the sources for retrieving the information that must be stored in the NPX files for each dataset.

**Table 2:** The sources for retrieving the information that must be stored in the NPX files.

**DataSet**	**Sensors**	**SR**	**Encoder**	**Events**	**ISI**	**Num. Iter**.	**Ref/Gnd**.	**True labels**	**Classif. Labels**
BCI comp 2	FTF	DP	DP	DF, FTF	DF*/DP	DP/SP	SP	DF/FTF	-
BCI comp 3	FTF	DP	DP	DF, FTF	DF*/DP	DP/SP	SP	DF/FTF	-
BNCI DS8	DF	SP	DP	DF	DF*/DP	DP/SP	SP	DF	-
BNCI DS9	DF	DF	DP	DF	DF*/DP	DP/SP	SP	DF	-
Akimpech	FTF	DF	DP	DF, FTF	DF*/DP	FTF	DP	DF/FTF	FTF
BTFC 180	DF	DF	DF/SP	DF	DF	DF*/SP	DF	DF	-
BTFC 250	DF	DF	DF/SP	DF	DF	DF*/SP	DF	DF	-
BTFC 800	DF	DF	DF/SP	DF	DF	DF*/SP	DF	DF	-
IEEE ERP	DF	DF	SP	DF	SP	SP	SP	DF	-
IEEE UVA	DF	DP	DP	DF	DP	DP	SP	DF*	-
LazyDog	FTF	DF	DF	DF	DF	DF	SP	DF	DF

^*^Indicates that the related information can be computed or deduced from the data file.

#### BCI competition 2, DataSet IIb

Signals of this dataset are available in MATLAB files, one per recording session, and made available for ML as described in Blankertz et al. (2004). The dataset was released for a BCI competition, where true labels of the signals in the calibration sessions were provided for competitors to classify the signals in other unlabeled sessions. The true labels were published after the winners of the competition were announced. It was then necessary to add the true labels to the converted files. Furthermore, it was crucial to compute, from the encoder and the flashed row or column, if stimulation was a target or a standard one.

A MATLAB script was then created to convert all the recordings sessions into NPX format. Some information was available in a separate DP, such as electrode names, sampling rate, the 6 × 6 matrix layout, the number of iterations, and the characters spelled during a copy task. Other information was described in the same DP but also deducible from other SPs. Signals were acquired from one subject during 19 recording sessions (11 for calibration). The subject was asked to spell a total of 72 characters among 12,960 provided stimuli in about 44 min. One of the recording sessions (the file: AAS011R06.MAT) presented one data packet loss, so this file was discarded. Spelled words belonged to an English dictionary, and also a 4-digit number was communicated.

#### BCI competition 3, DataSet III

Signals of this dataset were shared in 4 MATLAB files, one training (85 characters) and one testing (100 characters) file for each of the two subjects. They were released for a BCI competition (Blankertz et al., 2006) in a similar modality as the previous one. Thus, it was necessary to compute for each stimulus whether it was a standard or a target one. The format of these files was analogous but not identical to that relative to the competition described in subsection BCI competition 2, DataSet IIb. A MATLAB script was then written to convert the original files into NPX format. In a separate DP, available information included electrode names, sampling rate, the 6 × 6 matrix layout, the number of iterations, and the spelled characters. Subjects spelled a total of 380 characters, for which 66,600 stimuli were provided in more than 3 h and 20 min. All spelled words were a sequence of random characters without any meaning.

#### BNCI Horizon 2020, DataSet 8

This dataset was the only one selected for this study containing signals acquired from patients affected by amyotrophic lateral sclerosis (Riccio et al., 2013). The dataset is hosted by the BNCI Horizon 2020 repository (Brunner et al., 2015). One MATLAB file per subject was provided, but the data file omitted some relevant information, such as the sampling rate of the signals. This information was reported in a PDF document describing the paradigm. A MATLAB script was implemented to generate the NPX file from the original file after reading the PDF document and a SP. All patients had to spell the same 7 Italian words with a length of 4–5 characters and a number for a total of 280 characters and 33,600 delivered stimuli. The spelled characters were missing and had to be computed from the provided stimulation sequence, the nature (T or S) of each stimulus, and the encoder.

#### BNCI Horizon 2020, DataSet 9

This dataset was downloaded from the same BNCI Horizon 2020 repository and included signals from two P300 experiments. The experiments compared two different user interfaces and layouts (Aricò et al., 2014). Although the donors were from the same research group as those of the BNCI DataSet 8, the internal structure of the MATLAB files was different. Thus, a separate MATLAB script for DataSet 9 was created to convert it. Each of the 10 subjects had to spell the same 18 characters: 5 numbers and 13 letters, without any semantic meaning. A total of 180 characters were spelled, and 17,280 stimuli were delivered. As in the DataSet 8, the spelled characters were missing and had to be deduced from the encoder, the stimulation sequence, and the nature (T or S) of each stimulus. The stimulation sequence of the 18 characters and timing were the same for all subjects. As a side note, the dataset was then processed (Bianchi, 2018) as if the acquisition of the signals would occur simultaneously among the subjects. This was done to simulate an offline collaborative BCI experiment, in which signals from all subjects were aggregated together to generate desired outputs.

#### Akimpech

The Akimpech dataset (Ledesma Ramírez et al., 2010) included data from 30 volunteers. The signals of each subject were recorded during 4 sessions and stored in MATLAB files. The first session was to calibrate the BCI used in the successive recording, and each volunteer had to spell three words: ‘*calor'*, ‘*carino'*, and ‘*sushi'*. During the second session, the subject had just to spell the word '*sushi'*. During the third and fourth sessions, the subject was free to spell any word. The number of iterations was 15 for the first 3 sessions, which could be reduced to one single iteration per spelled word during the last session. Several pieces of information – such as the characters to be spelled, those classified, the number of iterations for each spelled word, the session number, and other details – were stored in a clear and self-documenting FTF. Thus, the information could be easily extracted from the file by a dedicated software tool after a programmer easily identified how to get access to the desired data. Sensor labels were also stored in a separate file. A MATLAB script was implemented for converting the data into NPX format. The entire dataset comprises 1,692 spelled characters and 227,679 delivered stimuli, making this study's second largest.

#### braINterface DataSets

The braINterface Lab released three datasets, each of them recorded from 10 subjects. The datasets were characterized by three different ISI: 180, 250, and 800 ms (Bianchi et al., 2010). Each subject had to select all 36 characters once in a different order. The number of iterations was always 15. Because the files were already available in NPX file format, no specific conversion was necessary.

#### IEEE DataPort, ERP-BCI

Signals of this dataset were available in a Python pickle format, a byte stream of python objects. The codes to convert the pickle format into a Python list were provided in a separate DP. The python list was retrieved by using the codes. The list had a total of 16 items, corresponding to 16 subjects. Each item was then converted into a NumPy array using the python NumPy library. Each array contained segmented signals of a subject with target/non-target labels, the sampling frequency at 128 Hz, the electrode names, and the total number of events. The signals of all 16 subjects were sorted into the target and non-target groups. A custom python script was developed to transform the array into a MATLAB format. Afterward, the MATLAB format was converted into the NPX format using a MATLAB script. According to the DP of the dataset, the signals of each subject were pre-processed and segmented into events, and each epoch was 600 ms long. The signals of the subject were collected *via* 16 electrodes and underwent pre-processing, including detrending, bad-channel removal, common-average referencing, bad-event eliminating, and band-pass filtering within 0–15 Hz.

The DP cited a SP (Abibullaev and Zollanvari, 2019) detailing the signal acquisition method. The SP was published based on signals of 7 subjects at the sampling frequency of 256 Hz. This sampling frequency was twice higher than that in the arrays of the 16 subjects obtained from the IEEE DataPort. The method utilized a 6 × 6 alphanumeric matrix as presented in the SP. The total number of target letters was 5, and each letter was repeated 5 times with 12 random flashes in each column and each row of the matrix. This yielded a total of 57,600 flashes. This number was reduced to 56,683 after bad-events elimination. The inter-stimulus interval (ISI) was 150 ms between two flashes, and each flash lasted 100 ms. Although the sequence of the flashes was unclear, the target-to-target interval (TTI) was 600 ms, as described in the SP. Nevertheless, it was unknown of the total length of the signal recording.

#### IEEE DataPort, GIB-UVA

All signals relative to 73 subjects and acquired in three different P300 Speller experiments with varying layouts were stored in a single 5.4 GB HDF file (Mason et al., 2010). The signals were pre-processed, segmented into 1,000 ms epochs starting from stimulus onset, and normalized in signal amplitude trial-by-trial. The normalization prevented thus computing averages from weighting each single evoked response in the same way. There were minor discrepancies between the stored information in the signals and that in scientific papers (Santamaría-Vázquez et al., 2019), such as the number of subjects involved in the experiments. A software tool in C++ programming language was implemented to extract the signals of one experiment, in which 22 subjects used the 6 × 6 matrix, to select letters and numbers. The framework of Microsoft Visual Studio 2022 was used in the tool implementation because libraries for handling HDF files are available (at https://www.hdfgroup.org/downloads/hdf5/). The large file size required a 64-bit compiler to handle it properly. The extraction generated 73 files, one per subject in the size of about 50 MB. However, only 22 of the files are related to a P300 Speller BCI compatible with the criteria of this study. It should be noted that because stimuli were provided in every 175 ms, each epoch shared the same 825-ms length with the previous and the next epochs. This implied a redundancy factor of close to 6. All signals of the experiment relative to our study could then be stored in less than 200 MB, about 4% of the original HDF file downloaded from the IEEE DataPort. The sequence of the stimuli was preserved, differing from the IEEE DataPort ERP-BCI dataset previously described. Finally, and for curiosity, the words to be copied were the names of characters from the Star Wars universe.

#### Lazy dog

**Figure 3:**
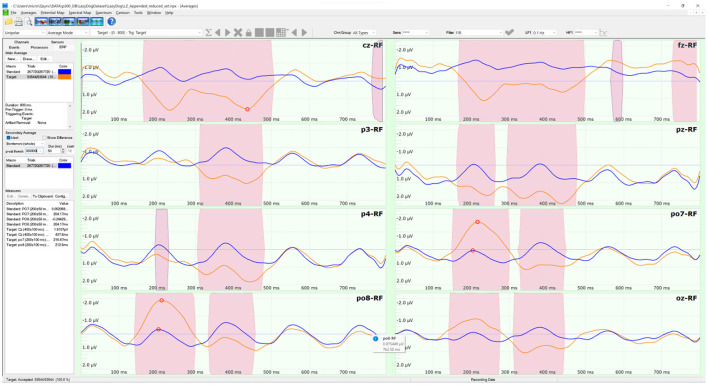
The ERP tool of the NPXLab Suite, depicting the grand averages of the LazyDog dataset with the 8 sensors selected for this study. The lines in orange are the averages over 53,544 target stimuli, while the lines in blue are those relative to the 267,720 non-target ones. Pink-shaded areas indicate that the difference between the two averages is statistically significant (*t-*test, *p* < 0.0001) after a Bonferroni correction for multiple comparisons.

The LazyDog dataset (Krusienski et al., 2008) holds 454 files in BCI2000 format (Schalk et al., 2004). The BCI2000 file format is well documented (https://www.bci2000.org/mediawiki/index.php/Technical_Reference:BCI2000_File_Format) and supported by freely available tools, including its native BCI2000Viewer. Besides sensor labels stored in a separate TXT file, the file format is the only one to contain all the information of a BCI paradigm, including the results of online classifications, this last is available also with the Akimpech dataset. The dataset was the largest among the selected datasets, with 1,798 spelled characters and 321,264 stimuli. The dataset involved 8 subjects, each performing up to 7 recording sessions. During each session, the subjects had to copy the sentence “*the quick brown fox jumped over XYZ lazy dog*”, where XYZ indicates a 3-digit random number. This sentence was inspired by the pangram “*the quick brown fox jumps over the lazy dog*”, which includes all the letters of the English alphabet and is often used by typographers. The second “the” of the pangram was substituted with a random 3-digit number in the copied sentence to allow the selection of every alphanumeric character of the symbols matrix. Two grand averages were computed on the 454 files of the 8 sensors selected for this study. The averages were distinct as the target (in orange) and non-target (in blue) stimuli, as illustrated in Figure 3. The pink-shaded areas of the averages indicated their significant differences in a sample-based *t*-test (*p* < 0.0001) after a Bonferroni correction for multiple comparisons.

### Implementation of software tools to retrieve and visualize P300 speller datasets

**Figure 4:**
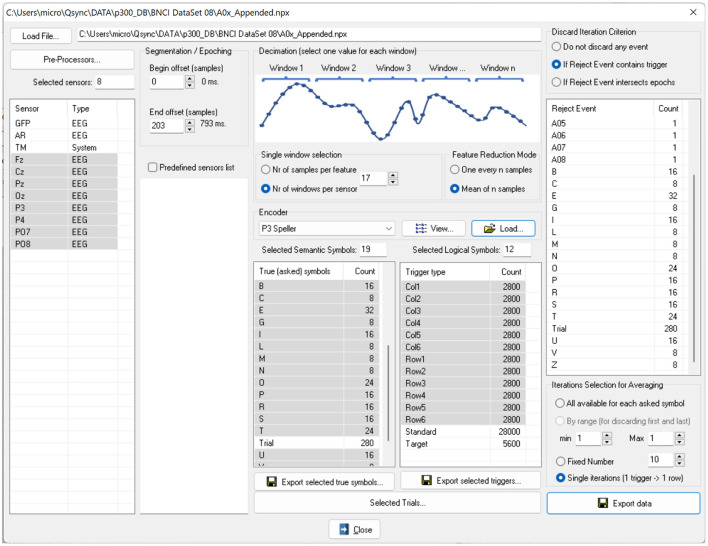
The user interface of the ERP Exporter software. Once a file is loaded, all controls are populated with sensor names, encoders, SSs and LSs. The LSs provide the triggers for segmenting signals. It is possible to undertake a feature reduction by decimating the signals, since the SSs represent the characters to be spelled and to average the signals over the same SS before exporting or saving them.

We implemented the ERP Exporter software and released it for researchers to test ML algorithms easily. The software allows ML experts to avoid being tangled with the details of the P300 paradigm. Since all information is already stored in NPX files, the experts don't have to read any external documents. The software's user interface is shown in Figure 4. Once a user of the software has loaded an NPX file, all software controls are populated with data extracted from the file. Specifically, sensors, SSs, LSs, and encoders allow the creation of the large features matrix and 4 column vectors as the following:

1) The X data matrix: this matrix can also be considered as a list of row vector features. Each feature vector is obtained by concatenating segmented signals (epochs or consecutive samples) from different sensors, time locked with a stimulus. The number of features is then determined by the selected sensors, the epoch size and some optional decimation strategies to average or skip consecutive samples. Decimation is implemented assuming that consecutive samples convey similar, and then redundant, information. The number of the matrix rows (i.e., the size of the list of features) depends on the way evoked responses are handled: they can be exported one by one (e.g., 180 rows vectors per spelled character for 15 iterations paradigms) or after averaging all iterations over the same spelled character, yielding 12 rows.
2) The Y label column vector: this vector contains the values 1 and −1 for target and non-target (standard) stimuli, respectively. The values are assigned based on the deduction of the true label for each stimulus because the NPX file contains the encoder, the LSs (stimuli), and the true SSs.
3) The I iteration column vector: this vector keeps the iteration index of each spelled character (e.g., a number between 1 and 15 for 15 iterations).
4) The C stimulus ID column vector: this vector holds the index of each stimulus (e.g., a number between 1 and 12 to identify the 6 rows and columns). This is necessary for the ML experts to know which stimulus was provided, regardless of its target or non-target nature.
5) The msg column vector: this vector maintains the characters that the user had or wanted to select.

The size of each column vector is the same as the number of rows of the X matrix. Other settings of the ERP Exporter are also available, and their effects on the data are described in the documentation released with the software.

Finally, the matrices and vectors can be exported in either ASCII or ARFF file format. The ARFF format is adopted by the Weka software, a collection of visualization tools and algorithms for data analysis and predictive modeling (Frank et al., 2016).

## Results

At the end of the entire process, more than 1,000 files were generated in the same NPX format. The files store all necessary information (i.e., features, labels, etc.) for training a classifier without the need to retrieve or read additional information from external documents. The generation of the files implies that the procedure of extracting the features and labels to train and test a classifier would last seconds instead of days or weeks, as required in present practice. Currently, researchers spend a lot of time understanding how data are stored and how to retrieve needed information and writing codes or MATLAB scripts to extract it. Adopting a standard file format reduces this need by eliminating it, allowing the use of existing software tools, or limiting it for processing specific datasets to meet the file format specifications.

The process involved EEG recordings referring to 127 subjects (including patients) and relative to more than 6,800 spelled characters. The total number of stimuli was 1,168,230, representing the largest set of BCI data for the P300 Speller based on a 6 × 6 alphanumeric matrix. The duration of the EEG recordings was estimated at more than 100 h. This data was approximated by defect since the two IEEE DataPort datasets have no details about their recording durations due to their provided signals stored in overlapping segments. These and other details are reported in Table 3.

**Table 3:** Summary of each dataset and its N200 and P300 latencies with relevant electrodes.

**DataSet**	**Open/closed loop**	**Subj**	**SR [Hz]**	**Sens**	**Chars**	**Flashes**	**ISI [ms]**	**Num Iter**	**N200 (PO7) [ms]**	**P300 (Cz) [ms]**
BCI Comp 2	Closed	1	240	61	72	12,960	175	15	279.2	329.2
BCI Comp 3	Closed	2	240	61	380	66,600	175	15	187.5	329.2
BNCI DS8	Closed	8	256	8	280	33,600	250	10	210.9	472.6
BNCI DS9	Closed	10	256	16	180	17,280	250	8	191.4	347.6
Akimpech	Closed	30	256	10	1,692	227,679	188	1-15	171.8	406.3
BTFC 180	Open	10	256	61	396	71,280	180	15	195.3	343.7
BTFC 250	Open	10	256	61	360	64,800	250	15	195.3	343.7
BTFC 800	Open	10	256	61	342	61,560	800	15	156.2	367.1
IEEE ERP	Closed	16	128	16	Unknown	56,683	150	Unknown	296.8	515.6
IEEE UVA	Closed	22	128	8	1,303	234,540	125-475	15	296.8	296.8
Lazy Dog	Closed	8	240	61	1,798	321,264	175	15	216.6	437.5

**Figure 5:**
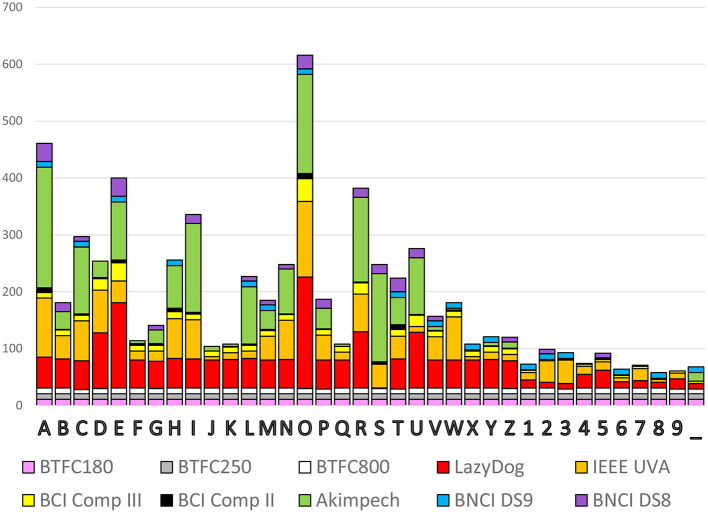
Cumulative occurrence of the 36 alphanumeric symbols to be selected with the contribution of each dataset.

Once converted to the same format, it is easy to analyze and compare data (i.e., evoked response latencies). In <15 min, we computed the occurrence of each spelled character to yield a histogram with the contribution of each dataset, as reported in Figure 5. The cumulative occurrence of each character was not uniform indeed, with the numbers less selected than the characters and the vowels more frequently selected than the consonants. Some datasets (e.g., the three BTCF) contributed uniformly across all 36 alphanumeric symbols, while others (e.g., Akimpech) had distinctly differentiated contributions.

**Figure 6:**
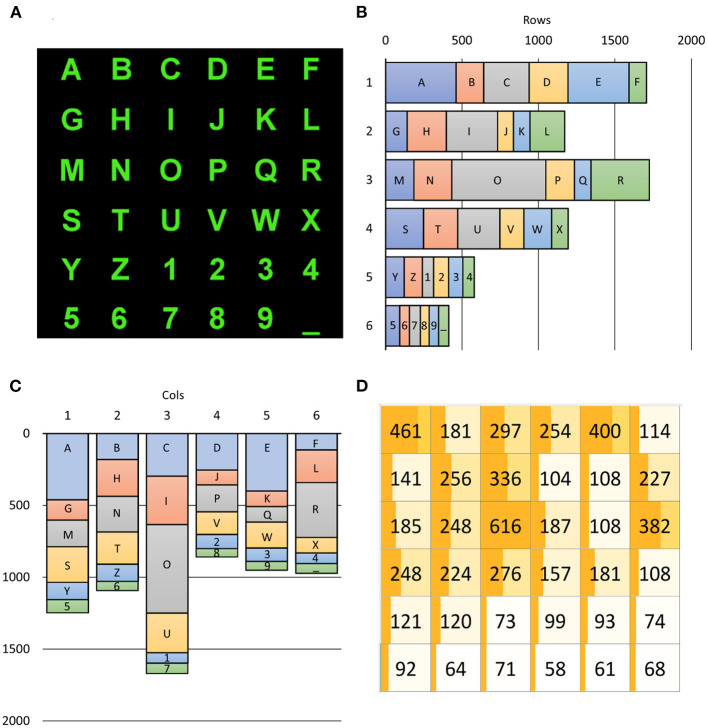
Alphanumeric symbols **(A)** and their contribution to target occurrences **(B–D)**. The **(B,C)** report the number of times a row, and a column includes the SS the user wanted to select, with the explicit contribution of each SS. The **(D)** reports a 6 × 6 matrix, matching that of **(A)**, in which the occurrence of each SS is shown: for example, the “R” character had to be selected 382 times across all 6,793 spelled ones (5.62%). The dark orange bar in each SS cell of **(D)** is proportional to the number of selections of the corresponding character as well as its background color, which is lighter for poorly selected characters. The darkness of the bar is proportional to its percentage. It is evident from **(B–D)** how unbalanced were the required users' selections.

Since the 36 alphanumeric symbols are selected with different probabilities, target rows and columns are also not equiprobable according to Figure 6. As shown in Figure 6B, the selection of rows was highly variable. The first and third rows were mostly selected (25.13 and 24.41%, respectively), while the last two rows – including the numbers – were rarely selected (8.54 and 6.09%). In contrast, the selection of columns (as illustrated in Figure 6C) varied much less, except for the third column, which is populated with three vowels (I, O, and U). The selection of the third column (24.57%) happened twice more frequently than the least selected fourth column (12.65%). Notwithstanding, further investigations are imperative to discover whether such unbalanced occurrences affect ML training procedures to impact the performances of BCI spellers.

Grand averages on each dataset were computed to calculate the latencies of the N200 and P300 components at the PO7 (for the N200) and Cz (for the P300) electrodes. The results of the calculation are reported in Table 3. Large variability was observed for both components, spanning from 156.2 to 279.2 ms for the N200 and from 296.8 to 515.6 ms for the P300. The variability might result from factors such as experimental setups, paradigm parameters, selected population, and pre-processing procedures. For example, an experimental setup can introduce disparate latencies at various stages, employ distinctive luminance and persistence of stimuli, or use a different acquisition setup (e.g., active vs. passive electrode, ground, reference location, etc.). Some paradigm parameters, like the ISI, can cause interference among the evoked responses. Finally, some datasets were pre-processed, whereas others did not. Thus, all these factors could contribute to the observed variability of the computation results.

All the above analyses and computations were completed in <1 h, demonstrating the great advantage of our entire process for time-saving. Moreover, there was no need for extra coding to perform the analyses and computations once signals were converted from their original formats to the NPX one.

## Discussion and conclusions

Having access to large datasets is advantageous as it allows researchers to improve the training of ML classifiers. In turn, the improved classifiers could enhance the usability of BCI systems, for example, by reducing the time needed to calibrate them. However, the current practice (as we experienced) of reading documents and converting each dataset can take an unnecessary huge amount of time and requires a certain degree of programming expertise. This limits potential users who cannot access the dataset without external support.

In this study, we proposed an entire process of merging datasets relative to the same BCI paradigm. The process was assessed using 10 different datasets, and each one was converted to the same file format to hold all the necessary information to perform the analyses and be extensible for future needs. In turn, it is required to convert datasets from their originals to a well-defined file format (NPX in this study). The conversion required a vast amount of time due to two factors. One factor was to write software in C, C++, Python, and MATLAB because the datasets were stored in different ways. Another factor was to read several papers and documentation since some information was available only in human-readable formats (e.g., a PDF file) or after inspecting the specifications of file formats. Once completing the conversion into the format, including all needed information to train a classifier (such as the IEEE P2731 Level 1 work-in-progress proposal), the analyses could be accomplished within 1 h – much faster than the days or weeks needed in current practice.

The lack of standards represents a great barrier for anyone who wants to compare or analyze evoked responses from different repositories. Herein, the IEEE Standards Association P2731 Working Group (WG) was established and is actively working toward providing the standards in various areas of BCI. Indeed, most authors of this study are active members of the WG, and the study represents a proof of concept of their vision for standardizing BCI data storage. Although a work in progress, a proposal for standardization is close to being completed by the WG.

For standardization, we identified the information that should be stored in a file for training a classifier. This study demonstrated the advantage of having a unique and well-defined file format for drastically reducing the time needed when analyzing multiple datasets. The demonstration sets a foundation for applying the format to data storage to facilitate the training and testing of new ML algorithms or enable access to new datasets. Notwithstanding, the adopted NPX format serves as an example of what information needs to be included in a standard BCI file, according to the FAIR principles, to enable processing signals without reading additional documentation. However, the format adopted in this study does not represent a proposal for a standard technical implementation because all stakeholders should be involved in its definition. It is the opinion of the IEEE P2731 WG that the definition of the included information is more critical and important than the technology used for storing them, providing that it could be easily extendable and that the additions do not break the backward compatibility with previously released tools.

The scope of this study is to convert datasets derived from the same P300 paradigm with identical matrix size, character layout, stimulation strategy (rows and columns), and description (e.g., which row and column are flashing). The conversion attempts to illustrate pitfalls of how the datasets are usually described and stored in the current practice. We observed huge differences, even if the data are hosted in the same repository or released by the same research group. Indeed, there are other valuable approaches to merge datasets from different sources, such as the MOABB project that dealt with various BCI paradigms and populated with more datasets than those included in this study. However, few of them contain the same information, even though many datasets are relative to P300 ERPs. It should be noted, for example, that even different matrix sizes may imply amplitude variations in the evoked responses. The variations might be negligible but need to be investigated in future work. In short, we aimed to minimize as much as possible differences among the datasets used in this study to demonstrate the creation of a large “uniform” dataset for facilitating future work.

We revealed two aspects to benefit users without programming expertise. One aspect is that the same software (e.g., the ERP Exporter) can be used to process all datasets. Another aspect is that it is sufficient to implement only once any not yet-available software tool for processing all files, including those released in the future, provided they are in a well-defined file format. For example, the ERP Exporter allows potential users to extract the necessary information to train a classifier in a few seconds without any external support or programming expertise.

Our experience analyzing all datasets listed in Table 1 prompts the following suggestions for releasing recordings into the public domain. We would suggest that sharing unprocessed datasets permits processing them in the same way. The processing and merging are feasible due to the wide availability of pre-processing capabilities for EEG and ERP signals. For the same reason, the signals should not be segmented into epochs because some epoch lengths might be too short to be analyzed (e.g., 600 ms for the IEEE ERP dataset, while several times at least 800 ms were analyzed). Signal normalization should also be avoided, which can have negative consequences on the procedure of averaging and can be performed later on. Moreover, the stimulation sequence provided to the user should be kept. Finally, we would suggest avoiding mixing experiments into a unique huge file, which might imply downloading unnecessary data.

Missing information, which we observed as relevant, is related to the stimulation sequence. Sometimes, just “target” and “standard” labels are provided or rearranged in an order different from the recording one after removing some noisy trials. This prevented us from knowing the spelled character on one side and the full set of stimuli that were provided to select one single character on another side.

In summary, we found several difficulties during this study of file conversion, mainly due to the fact that human readers are needed to extract pieces of information in separate documents (usually PDF files) for the conversion. Table 2 summarizes the sources to extract the necessary information that falls chiefly into 4 categories: Data Files, Descriptive Papers, Formatted Text Files, and Scientific Publications.

As a proof-of-concept, this study represents the beginning of many forthcoming activities: to explore new transfer learning strategies, to compare different classifiers, to invent novel pre-processing methods, etc. The outcomes of the study – the largest set of BCI data for the P300 Speller with over 1,168,230 stimuli – could provide a base of undertaking such exploration, comparison, and invention in a new way other than those used before. Moreover, the set can be extended whenever compatible datasets are released into the public domain. The extensibility of the set would encourage BCI researchers to develop and release software tools that could be utilized by others and applied to a huge amount of data. The more software tools and data are available, the more feasible it is to drastically boost BCI progress in bridging gaps between computational intelligence and neurosciences.

## Data availability statement

The datasets presented in this study can be found in online repositories. The names of the repository/repositories and accession number(s) can be found in the article/supplementary material.

## Ethics statement

The studies involving human participants were reviewed and approved by several local Ethical Committees, one for each of the shared datasets, described in various publications. We have not acquired new data for this work. The patients/participants provided their written informed consent to participate in this study.

## Author contributions

NZ and YH converted the original data format (pickle) of the IEEE-ERP dataset (i.e., P300 BCI) that came from the IEEE DataPort into the MATLAB format, and contributed to writing and editing the manuscript. LB converted datasets into NPX from various sources, implemented the ERP Exporter, updated the NPXLab Suite to support IEEE P2731 specifications, and contributed to writing the manuscript. RF contributed in datasets searching and collection, metadata retrieval, and data compatibility check. GS-A conceptualized the study and contributed to writing and editing the manuscript. All authors contributed to the article and approved the submitted version.
